# The prognostic values of CYP2B6 genetic polymorphisms and metastatic sites for advanced breast cancer patients treated with docetaxel and thiotepa

**DOI:** 10.1038/srep16775

**Published:** 2015-11-25

**Authors:** Qingkun Song, Xinna Zhou, Jing Yu, Ningning Dong, Xiaoli Wang, Huabing Yang, Jun Ren, H Kim Lyerly

**Affiliations:** 1 Beijing Key Laboratory of Therapeutic Cancer Vaccines, Beijing Shijitan Hospital, Capital Medical University Cancer Center, 10 Tieyi Road, Beijing 100038, China; 2 Department of Medical Oncology, Peking University Cancer Hospital & Institute. 52 Fucheng Rd, Beijing 100142, China; 3 Department of Digestive Diseases, Beijing Friendship Hospital, Capital Medical University, Yongan Road 95, Beijing 100050, China; 4 Department of Surgery, Duke University Medical Center, 203 Research Drive, Suite 433, Box 2606, Durham, NC 27710, United States

## Abstract

This study investigated interactive effects of CYP2B6 genotypes and liver metastasis on the prognosis of metastatic breast cancer patients who received combined chemotherapy of docetaxel and thiotepa. Totally 153 patients were retrospectively genotyped rs8192719 (c.1294 + 53C > T) and rs2279343 (c.785A > G). Kaplan-Meier method and Cox Proportional Hazard Regression model were used to estimate the survival. Patients with liver metastasis had worsen prognosis, conferring a 2.26-fold high risk of progression and 1.93-fold high risk of death (*p* < 0.05). Both CT/TT genotype of rs8192719 (c.1294 + 53C > T) and AG genotype of rs2279343 (c.785A > G) prolonged survival (*p* < 0.05). Furthermore, among liver metastatic patients, AG genotype of rs2279343 (c.785A > G) was associated with a 47% reduced risk of death and a 6-month-longer overall survival (*p* < 0.05). Among non-liver metastatic patients, hazard ratios of CT/TT genotype of rs8192719 (c.1294 + 53C > T) were 0.45 for progression and 0.40 for death; and the corresponding survival was improved by 6 months and 16 months, respectively (p < 0.05). Genotypes of CYP2B6 had an interaction with clinical efficacy of docetaxel and thiotepa on metastatic breast cancer patients; and metastatic sites also affected clinical responses. Further therapies should take into account of chemotherapy regimen, genotypes of metabolizing enzymes and metastatic sites for the particular subpopulation.

Metastasis has become the leading causes for death among breast cancer patients. More and more evidence has supported that brain, liver and lung metastases are lethal. The apparent variations in clinical responses among metastatic breast cancer (MBC) patients treated with various salvage chemotherapy remain unclear^1^,^2^. We have explored specific chemotherapeutics of docetaxel and thiotepa [targeting liver metastasis (LM)] in MBC patients who had developed resistance to previous anthracycline and paclitaxel. It was believed that docetaxel plus thiotepa were efficient for liver metastasis^3^. Thiotepa could be rapidly metabolized by cytochrome P450 (CYP450) into its main metabolite *tepa* through oxidative desulfuration and generation of regional cytotoxicity in metastatic liver lesions^4^. We therefore recommended that docetaxel plus thiotepa could be optimally recommended as specific targeted-therapy for liver metastasis^3^. More recently, we have found the new biological activities of thiotepa in killing breast cancer stem cells^5^. Since CYP2B6 is a phase I xenobiotics metabolizing enzyme, a member of the cytochrome P450 superfamily^6^ capable of catalyzing the reaction of thiotepa, we postulated the genetic effects of single nucleotide polymorphisms (SNPs) on clinical outcome of MBC individuals treated with docetaxel and thiotepa. Two SNPs at rs8192719 (c.1294 + 53C > T) and rs2279343 (c.785A > G) in CYP2B6 gene were targeted and explored the clinical efficacy^7^,^8^. CT/TT genotype of rs8192719 (c.1294 + 53C > T) and AG genotype of rs2279343 (c.785A > G) led to a reduced metabolizing activity of CYP2B6 enzyme^9^. The primary endpoint of this study was the interactions of CYP2B6 genotypes with metastatic sites on the prognosis for MBC.

## Patients and Methods

### Selection of Patients

To be eligible for this study, patients must meet the following criteria: female, aged ≥18 years, histological proven MBC, previous treatment with anthracycline and paclitaxel, no previous treatment with docetaxel or thiotepa, Eastern Cooperative Oncology Group (ECOG) performance status of 0–2, at least one measurable lesion, adequate hepatic (serum bilirubin ≤1.5× upper normal limit and aspartate aminotransferase and alanine aminotransferase ≤1.5× upper normal limit), renal (serum creatinine ≤1.5× upper normal limit), and bone marrow (neutrophils ≥1.5 × 10^3^/μl, platelets ≥100 × 10^3^/μl, and hemoglobin ≥10 g/dl) functions, normal electrocardiogram, expected life expectancy of more than 3 months, no pregnancy or lactation, no serious or uncontrolled concurrent medical illness, and no history of other malignancies. Chemotherapy, radiotherapy, and hormonal therapy were all permitted provided that 4 weeks had passed prior to the study.

This study, which was registered at ClinicalTrials.gov (NCT01199393), was approved by ethnic committee of Peking University Cancer Hospital. From Aug 31 2010 to Dec 31 2014, each patient who participated in the study had signed an informed consent. Totally, 153 patients were recruited eligibly into the study and detected polymorphisms of CYP2B6. The experiments were performed in accordance with the approved guideline and regulation.

### Therapeutic protocols

This was an open-label and prospective study^3^. Docetaxel 35 mg/m^2^ was administered in 100 ml of 0.9% sodium chloride over 30 minutes, followed by thiotepa 30 mg/m^2^ in 250 ml of 0.9% sodium chloride over 30 minutes. Both drugs were given intravenously on days 1 and 8, every 21 days for two cycles at least. Premedication of dexamethasone 7.5 mg by mouth twice daily was administered on the day before docetaxel infusion and continued for a total of 3 days. 5-hydroxytryptamine-3 receptor antagonists were systematically administered to prevent emesis. Patients were scheduled to receive a maximum of 8 cycles, and chemotherapy was stopped in case of disease progression, patient refusal, or unacceptable toxicity. Adverse events were classified according to National Cancer Institute Common Toxicity Criteria (NCI-CTC) version 3.0^10^.

### Genotyping of CYP2B6 genetic polymorphisms

Genomic DNA was extracted from venous blood samples (4 ml) drawn before drug administration, using standard phenol-chloroform method. Polymorphism of rs8192719 (c.1294 + 53C > T) was genotyped by matrix-assisted laser desorption/ionization time-of-flight (MALDI-TOF) mass spectrometry method (Sequenom, Inc., San Diego) and rs2279343 (c.785A > G) was genotyped by polymerase chain reaction (PCR) and direct sequencing^11^. All samples were processed and analyzed by Beijing Genome Institute (BGI). To verify the results, 5% of the DNA samples were randomly selected for duplicate assays. All the results were generated and analyzed by laboratory staff unaware of patient status.

### Statistical Analysis

All data were analyzed by SPSS for windows version 15.0 (SPSS Inc., Chicago, IL, USA). Progression-free survival (PFS) and overall survival (OS) were estimated by Kaplan-Meier method. In the univariate analyses, the categorical variables between metastatic status and variant genotypes were analyzed by Chi-square test and the continuous variables were analyzed by t-test. The variables included age, estrogen receptor (ER) (negative vs. positive), progestin receptor (PR) (negative vs. positive), human epidermal growth factor receptor-2 (HER2) (negative vs. positive), menopausal status (pre- vs. post-), and chemotherapy (1^st^, 2^nd^, vs. ≥ 3^rd^ line).

The Kaplan-Meier survival curve of PFS and OS were estimated for liver metastasis and genotypes with log-rank tests. The significant variables in log-rank tests were further analyzed by Cox Proportional Hazard Regression Models with confounder adjustment (age, menopause, ER, PR, HER2 status and chemotherapy). All tests were two-tailed and the significant level was a *p* value of ≤0.05.

## Results

### Patients’ Characteristics

During the follow-up period, 134 patients had disease progressed and 100 patients died. 65 patients had LM and 88 patients had metastases involved other organs. The mean age of MBC patients with LM was 51.1 years old, 4 years younger than the other metastatic patients (*p* < 0.05) (Table 1). Between the patients with liver and other sites metastases, the frequencies of menopausal, ER, PR, HER2 status and molecular subtypes as well as chemotherapy were similar (*p* > 0.05) (Table 1). Between genotypes of rs8192719 (c.1294 + 53C > T) and rs2279343 (c.785A > G), all demographic and clinical factors distributed similarly (*p* > 0.05) (Table 2).

### Survival

**Figure 1:**
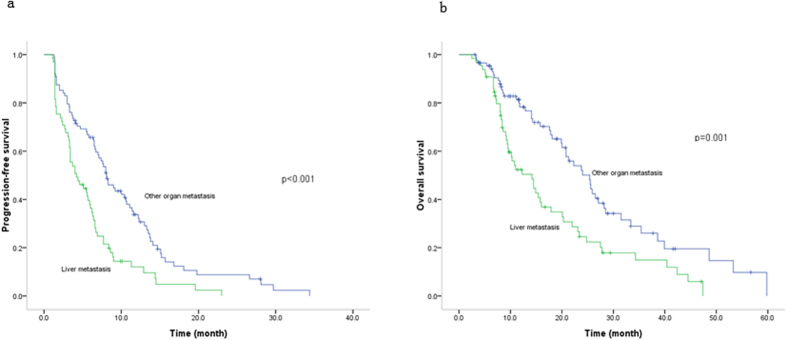
Effect of liver metastasis on MBC survival, (a) progression-free survival, (b) overall survival. Liver metastasis decreased the progression-free and overall survival.

The LM patients had a shorter survival than those with other metastatic sites (Fig. 1). PFS was 4.20 months in LM patients, nearly 4-month shorter than other metastatic patients and the HR of LM was 2.26 (95% CI, 1.53–3.36) (Table 3). OS of LM patients was 14.20 months, in contrast with 25.40 months in other metastatic patients (Table 3). LM produced a 93% increased risk for death (HR = 1.93, 95% CI, 1.26–2.95) (Table 3).

**Figure 2:**
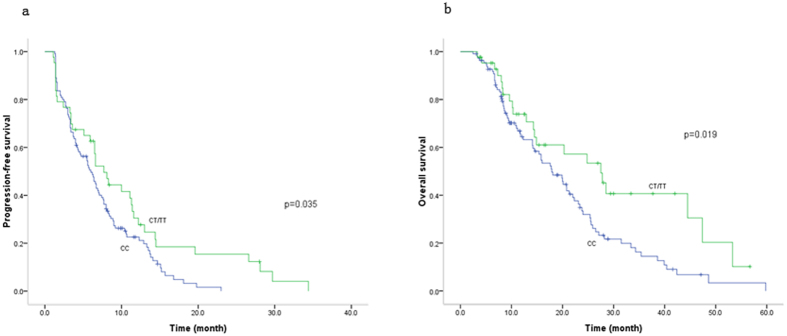
Genotypes of rs8192719 (c.1294 + 53C > T) and the survival of MBC, (a) progression-free survival, (b) overall survival. CT/TT genotype of rs8192719 (c.1294 + 53C > T) had longer progression-free and overall survival than CC genotypes.

The CT/TT genotypes of rs8192719 (c.1294 + 53C > T) introduced longer survival time than the CC genotype in MBC patients (Fig. 2). PFS of CT/TT genotypes was 7.70 months, 1.7-month longer than CC genotype, and the HR of CT/TT genotypes was 0.62 for progression (95% CI, 0.50–0.94) (Table 3). Patients with CT/TT genotypes of rs8192719 (c.1294 + 53C > T) had the OS of 27.50 months compared to 17.90 months in CC genotype (*p* < 0.05); HR of CT/TT genotypes for death reduced to 0.52 (95% CI, 0.32–0.85) (Table 3).

**Figure 3:**
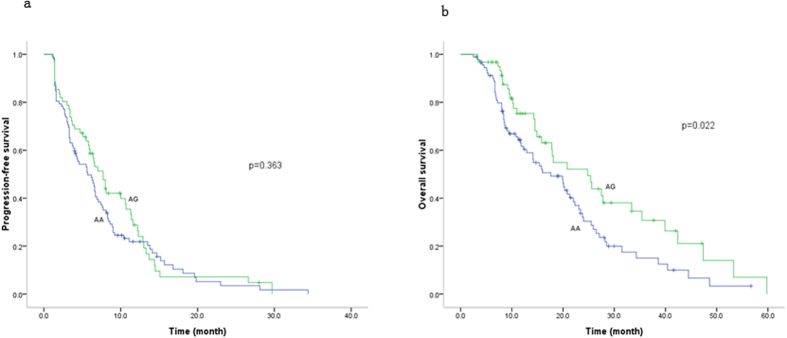
Genotypes of rs2279343 (c.785A > G) and the survival of MBC, (a) progression-free survival, (b) overall survival. The overall survival was longer in AG genotype and AA genotype.

The AG genotype of rs2279343 (c.785A > G) produced a longer OS than the AA genotype (Fig. 3). OS in patients harboring the AG genotype was 24.80 months, 12-month longer than the AA genotype (*p* < 0.05); the corresponding risk for death was reduced by 46% (HR = 0.54, 95% CI, 0.35–0.84) (Table 3).

### Interactive effects of LM and SNPs

PFS was similar between the CT/TT and CC genotypes of rs8192719 (c.1294 + 53C > T) among LM patients, however, among other metastatic patients, CT/TT genotypes had a PFS of 11.40 months, 3.8 month longer than CC genotype; and a 55% reduced risk of progression (HR = 0.45, 95% CI, 0.24–0.86) (Table 4). OS of other organ metastasis patients with CT/TT genotypes of rs8192719 (c.1294 + 53C > T) was 7.4 month longer than CC genotype, with a HR of 0.40 (95% CI, 0.17–0.91) for death (Table 5). OS in LM patients with the AG genotype of rs2279343 (c.785A > G) was longer than the AA genotype (14.90 vs. 9.40 months), and related with a death risk reduced by 47% (95% CI, 0.29–0.98) (Table 5).

## Discussion

The emerging concept of precise and personalized medicine has been recently proposed world widely, which enables us to apply chemotherapeutic protocols tailored with individual genetic identity. Significant heterogeneity in drug response can be observed in the clinical practice. Unfortunately, there is no reliable biomarker to help to select patients who are most likely to benefit from the specific treatment whereas save others from unnecessary toxicities produced by ineffective treatments. Although factors such as age, organ functions and tumor biology can explain different drug responses to a certain extent, genetic constitutions are reported to account for 20% to 95% of this variability^12^,^13^. A growing number of literatures suggest that genetic polymorphisms (e.g., SNPs) in metabolizing enzymes may be a major determinant of drug response^14^. In addition, SNPs in CYP450 may play a critical role since the enzyme is involved in metabolisms of about 80% of all phase I drugs^15^. Therefore, it is reasonable to hypothesize that those SNPs in CYP450 influence clinical outcome of docetaxel/thiotepa regimen. Our previous study on docetaxel plus capecitabine versus docetaxel plus thiotepa as ≥2^nd^-line chemotherapy in the MBC patients showed similar clinical responses and tolerable toxicities^3^.

Our present study indicated that docetaxel plus thiotepa was effective and safe for MBC patients, with the median PFS of 6.5 months, and median OS of 20.0 months. It was comparable to other docetaxel-containing regimens for MBC^16^,^17^,^18^,^19^,^20^. Interestingly, liver metastatic patients had reduced PFS and OS than other metastatic patients, and the poorer survival of liver metastasis was presented from previous studies^21^,^22^. Liver has been appointed as the centralized site responsible for maintain the physiologically drug metabolism to deliver the active compound into the circulation. Liver function detained from hepatocytes from extraordinary toxic exposures, Patients who appeared by a lowered albumin and an elevated bilirubin levels ignited the onset of liver failure, leading to a poor clinical outcome^22^. Decompensation of liver function was observed to be related with a poor outcome among MBC patients regardless of any exposures to treatments^23^. Metastasis damaged liver function and produced a poor survival among the patients.

Docetaxel is a well-established anti-mitotic medication used mainly for the treatment of breast cancer for more than decade^24^. Thiotepa is a traditionally alkylating antineoplastic agent used for breast cancer and bladder cancer etc^25^. The data from our group showed the combination of docetaxel with thiotepa has been reported to obtain an optimistic outcome in MBC patients^26^. CYP2B6 is a phase I xenobiotics metabolizing enzyme and a member of the cytochrome P450 superfamily^6^. CYP2B6 as a monooxygenase, participates in drug metabolism and synthesis of some lipids^6^. The enzyme metabolizes several anti-cancer drugs, such as cyclophosphamide and thiotapa^4^,^6^. CYP2B6 first metabolizes thiotepa to tapa, and then phase II drug-metabolizing enzymes (GSTA1/P1) catalyze tepa to monoglutathionlytepa^4^. CT/TT genotypes of rs8192719 (c.1294 + 53C > T) and AG genotype of rs2279343 (c.785A > G) caused a decreased metabolizing activity of CYP2B6^9^. A low metabolizing activity of CYP2B6 resulted in a high exposure to thiopeta and tepa^4^,^9^. The decreased activity of drug-metabolizing enzyme was possible to introduce an increase in drug exposure, and then an optimistic survival was observed among patients of CT/TT genotypes of rs8192719 (c.1294 + 53C > T) and AG genotype of rs2279343 (c.785A > G).

Due to the metabolism properties of thiotepa which could be converted into tepa in the liver, we were interested in testifying the feasibility of applying a specific chemotherapeutic regimen for patients with liver metastases. In this study, AG genotype of rs2279343 (c.785A > G) was associated with longer OS among liver metastatic MBC patients, however, CT/TT genotype of rs8192719 (c.1294 + 53C > T) was associated with longer PFS and OS among non-liver metastatic MBC patients. The docetaxel plus thiotepa regimen had a better efficacy to patients with AG genotype of rs2279343 (c.785A > G) and liver metastasis, as well as the patients with the CT/TT genotype of rs8192719 (c.1294 + 53C > T) and metastases to organs other than liver.

## Conclusions

The regimen of combined docetaxel and thiotepa achieved a better prognosis among MBC patient harboring AG genotype of rs2279343 (c.785A > G) and CT/TT genotype of rs8192719 (c.1294 + 53C > T). Liver metastasis conferred a worse survival than metastases other than liver. Under docetaxel and thiotepa regimen, AG genotype of rs2279343 (c.785A > G) correlated with a better prognosis among liver metastatic patients, whereas CT/TT genotype of rs8192719 (c.1294 + 53C > T) favored other-than-liver metastatic patients. The chemotherapy regimen, together with genetic polymorphisms of drug-metabolizing enzymes as well as metastatic sites should be taken into account in precise medicine.

## Additional Information

**How to cite this article**: Song, Q. *et al.* The prognostic values of CYP2B6 genetic polymorphisms and metastatic sites for advanced breast cancer patients treated with docetaxel and thiotepa. *Sci. Rep.* **5**, 16775; doi: 10.1038/srep16775 (2015).

## Figures and Tables

**Table 1:** Characteristics of MBC patients between liver and other metastasis.

	Metastasis		*p*
	Liver metastasis	Other metastasis	
Age, years (mean ± SD)	51.1 ± 9.87	55.3 ± 10.69	0.014
Menopause, n (%)			0.180
Post-menopause	43 (66.2)	68 (77.3)	
Pre-menopause	22 (33.8)	20 (22.7)	
ER, n (%)			0.741
Positive	42 (65.6)	53 (61.6)	
Negative	22 (34.4)	33 (38.4)	
PR, n (%)			0.813
Positive	39 (60.9)	49 (57.6)	
Negative	25 (39.1)	36 (42.4)	
HER2, n (%)			0.469
Positive	12 (18.8)	11 (13.3)	
Negative	52 (81.3)	72 (86.7)	
Molecular subtypes, n(%)			0.426
Luminal A	46(71.9)	46(71.9)	
Luminal B	0(0)	1(1.6)	
HER2+	5(6.0)	5(7.8)	
Triple negative	23(27.4)	12(18.8)	
Chemotherapy, n (%)			0.469
1^st^line	29 (44.6)	42 (47.7)	
2^nd^ line	21 (32.3)	31 (35.2)	
≥3^rd^ line	15 (23.1)	15 (17.0)	

**Table 2:** Associations of MBC characteristics with CYP2B6 genotypes.

	rs8192719 (c.1294 + 53C > T)			rs2279343 (c.785A > G)		*p*
	CC	CT/TT	*p*	AA	AG	
Age, years (mean ± SD)	53.6 ± 10.36	53.4 ± 11.05	0.905	53.9 ± 10.15	53.0 ± 11.13	0.577
Menopause, n (%)			0.198			0.308
Post-menopause	83 (75.5)	28 (65.1)		70 (76.1)	41 (67.2)	
Pre-menopause	27 (24.5)	15 (34.9)		22 (23.9)	20 (32.8)	
ER, n (%)			0.3			0.323
Positive	65 (60.7)	30 (69.8)		53 (59.6)	42 (68.9)	
Negative	42 (39.3)	13 (30.2)		36 (40.4)	19 (31.1)	
PR, n (%)			0.884			0.873
Positive	63 (59.4)	25 (58.1)		51 (58.0)	37 (60.7)	
Negative	43 (40.6)	18 (41.9)		37 (42.0)	24 (39.3)	
HER2, n (%)			0.43			0.182
Positive	18 (17.1)	5 (11.9)		17 (19.5)	6 (10.0)	
Negative	87 (82.9)	37 (88.1)		70 (80.5)	54 (90.0)	
Molecular subtypes, n(%)			0.768			0.454
Luminal A	71(67.0)	31(73.8)		59(67.0)	43(71.7)	
Luminal B	1(0.9)	0(0)		1(1.1)	0(0)	
HER2+	7(6.6)	3(7.1)		8(9.1)	2(3.3)	
Triple negative	27(25.5)	8(19.0)		20(22.7)	15(25.0	
Chemotherapy, n (%)			0.416			0.416
1^st^line	49 (44.5)	22 (51.2)		40 (43.5)	31 (50.8)	
2^nd^ line	38 (24.5)	14 (32.6)		35 (38.0)	17 (27.9)	
≥3^rd^ line	23 (20.9)	7 (16.3)		17 (18.5)	13 (21.3)	
Metastatic sites			0.529			0.845
Liver	45 (40.9)	20 (46.5)		38 (41.3)	27 (44.3)	
Other	65 (59.1)	23 (53.5)		54 (58.7)	34 (55.7)	

**Table 3:** Association of liver metastasis and CYP2B6 genotypes with survival.

Survival	Factors	MST (months)	*P*	HR (95%CI)	HR (95%CI)^a^
PFS	Other metastasis	8.10	<0.001	1.00 (ref.)	1.00 (ref.)
	Liver metastasis	4.20		2.01(1.41–2.86)	2.26 (1.53–3.36)
OS	Other metastasis	25.40	0.001	1.00 (ref.)	1.00 (ref.)
	Liver metastasis	14.20		1.95(1.31–2.91)	1.93 (1.26–2.95)
PFS	rs8192719 (CC)	6.00	0.035	1.00 (ref.)	1.00 (ref.)
	rs8192719 (CT/TT)	7.70		0.65(0.44–0.98)	0.62^b^(0.50–0.94)
OS	rs8192719 (CC)	17.90	0.019	1.00 (ref.)	1.00 (ref.)
	rs8192719 (CT/TT)	27.50		0.57(0.35-0.92)	0.52^b^ (0.32-0.85)
PFS	rs2279343 (AA)	5.70	0.363	1.00 (ref.)	1.00 (ref.)
	rs2279343 (AG)	7.70		0.85(0.60–1.21)	0.88^b^ (0.61–1.28)
OS	rs2279343 (AA)	17.60	0.022	1.00 (ref.)	1.00 (ref.)
	rs2279343 (AG)	24.80		0.62(0.40–0.94)	0.54^b^ (0.35–0.84)

^a^adjusted age, ER, HER2 and chemotherapy.

^b^further adjusted metastasis. MST, median survival time.

**Table 4:** Interactive effects of liver metastasis and CYP2B6 genotypes on progression-free survival.

Metastasis	Factors	MST (months)	*p*	HR (95% CI)	HR (95% CI)^a^
Liver	rs8192719 (CC)	4.40	0.928	1.00 (ref.)	1.00 (ref.)
	rs8192719 (CT/TT)	3.40		0.98(0.56–1.71)	1.07 (0.58–1.99)
Other	rs8192719 (CC)	7.60	0.011	1.00 (ref.)	1.00 (ref.)
	rs8192719 (CT/TT)	11.40		0.47(0.26–0.85)	0.45 (0.24–0.86)
Liver	rs2279343 (AA)	3.40	0.399	1.00 (ref.)	1.00 (ref.)
	rs2279343 (AG)	5.50		0.80(0.47–1.36)	0.76 (0.43–1.33)
Other	rs2279343 (AA)	7.60	0.629	1.00 (ref.)	1.00 (ref.)
	rs2279343 (AG)	10.60		0.89(0.55–1.43)	0.83 (0.48–1.43)

^a^adjusted age, ER, HER2 and chemotherapy. MST, median survival time.

**Table 5:** Interactive effects of liver metastasis and CYP2B6 genotypes on overall survival.

Metastasis	Factors	MST (months)	*p*	HR (95% CI)	HR (95%CI)^a^
Liver	rs8192719 (CC)	11.00	0.150	1.00 (ref.)	1.00 (ref.)
	rs8192719 (CT/TT)	14.90		0.64(0.34–1.19)	0.65 (0.34–1.24)
Other	rs8192719 (CC)	22.30	0.013	1.00 (ref.)	1.00 (ref.)
	rs8192719 (CT/TT)	53.30		0.37(0.16–0.84)	0.40 (0.17–0.91)
Liver	rs2279343 (AA)	9.40	0.038	1.00 (ref.)	1.00
	rs2279343 (AG)	14.90		0.54(0.30-0.98)	0.53 (0.29–0.98)
Other	rs2279343 (AA)	22.30	0.102	1.00 (ref.)	1.00 (ref.)
	rs2279343 (AG)	33.40		0.60(0.32-1.11)	0.52 (0.27–1.01)

^a^adjusted age, ER, HER2 and chemotherapy.MST, median survival time.
